# The contribution of PA-X to the virulence of pandemic 2009 H1N1 and highly pathogenic H5N1 avian influenza viruses

**DOI:** 10.1038/srep08262

**Published:** 2015-02-05

**Authors:** Huijie Gao, Yipeng Sun, Jiao Hu, Lu Qi, Jinliang Wang, Xin Xiong, Yu Wang, Qiming He, Yang Lin, Weili Kong, Lai-Giea Seng, Honglei Sun, Juan Pu, Kin-Chow Chang, Xiufan Liu, Jinhua Liu

**Affiliations:** 1 Key Laboratory of Animal Epidemiology and Zoonosis, Ministry of Agriculture, College of Veterinary Medicine and State Key Laboratory of Agrobiotechnology, China Agricultural University, Beijing, China; 2 Animal Infectious Disease Laboratory, School of Veterinary Medicine, Yangzhou University, Yangzhou, Jiangsu Province, China; 3 School of Veterinary Medicine and Science, University of Nottingham, Sutton Bonington Campus, United Kingdom

## Abstract

PA-X is a novel protein encoded by PA mRNA and is found to decrease the pathogenicity of pandemic 1918 H1N1 virus in mice. However, the importance of PA-X proteins in current epidemiologically important influenza A virus strains is not known. In this study, we report on the pathogenicity and pathological effects of PA-X deficient 2009 pandemic H1N1 (pH1N1) and highly pathogenic avian influenza H5N1 viruses. We found that loss of PA-X expression in pH1N1 and H5N1 viruses increased viral replication and apoptosis in A549 cells and increased virulence and host inflammatory response in mice. In addition, PA-X deficient pH1N1 and H5N1 viruses up-regulated PA mRNA and protein synthesis and increased viral polymerase activity. Loss of PA-X was also accompanied by accelerated nuclear accumulation of PA protein and reduced suppression of PA on non-viral protein expression. Our study highlights the effects of PA-X on the moderation of viral pathogenesis and pathogenicity.

Influenza A virus (IAV) remains a significant threat to public health and causes great economic losses globally. Its pathogenicity is dependent on virus strain and host species, ranging from asymptomatic to 100% lethality. IAV is an enveloped negative-strand RNA virus, with eight genomic viral RNA (vRNA) segments. Originally, the influenza viral genome was thought to encode the following 10 viral proteins: PB2, PB1, PA, HA, NP, NA, M1, M2, NS1 and NS2^1^,^2^. Lately, additional novel proteins encoded from PB1 and PA mRNA have been discovered^3^,^4^,^5^. It was recently demonstrated that segment 3 of the influenza A virus encodes not only the PA protein, but also an additional novel protein, PA-X, which is translated as a +1 frameshift open reading frame (X-ORF) extension of a growing PA polypeptide^6^. PA-X inhibits host protein synthesis, contributing to host-cell shut down and inhibition of host antiviral response^6^,^7^. Moreover, PA-X modulates host inflammation, immune response, apoptosis, cell differentiation, and tissue remodeling^6^. Absence of PA-X protein expression in the pandemic 1918 H1N1 virus increased pathogenicity in mice^6^. However the role of PA-X proteins in current epidemiologically important influenza A virus strains are not known.

The 2009 pandemic H1N1 (pH1N1) influenza virus infection was the first pandemic of the 21st century and is still circulating in humans and pigs^8^,^9^,^10^. The highly pathogenic avian influenza (HPAI) H5N1 viruses have undergone widespread geographical expansion among wild and domestic birds and are highly lethal in chickens and humans, and may cause future influenza pandemics^11^,^12^,^13^. Comprehensive genetic analysis has shown that PA-X gene was conserved in all available influenza viruses including pH1N1 and H5N1 strains^6^,^14^. The PA-X ORFs of avian H5N1 influenza viruses are the full-length 61 amino acids in the X domain, while pH1N1 viruses express a truncated PA-X with 41 amino acids in the X domain.

In this study, we examined the role of PA-X on the virulence of pH1N1 and H5N1 influenza viruses and explored its possible role in the underlying processes including cell death, viral polymerase activity and changes of PA protein function. We showed that loss of PA-X increased viral replication and pathogenicity for both pH1N1 and H5N1 influenza viruses. Absence of PA-X enhanced viral polymerase activity, PA mRNA and protein expression, and reduced the virus-induced suppressive effect of protein synthesis.

## Results

### Generation of PA-X deficient pH1N1 and H5N1 (pH1N1-FS and H5N1-FS) viruses

**Figure 1:**
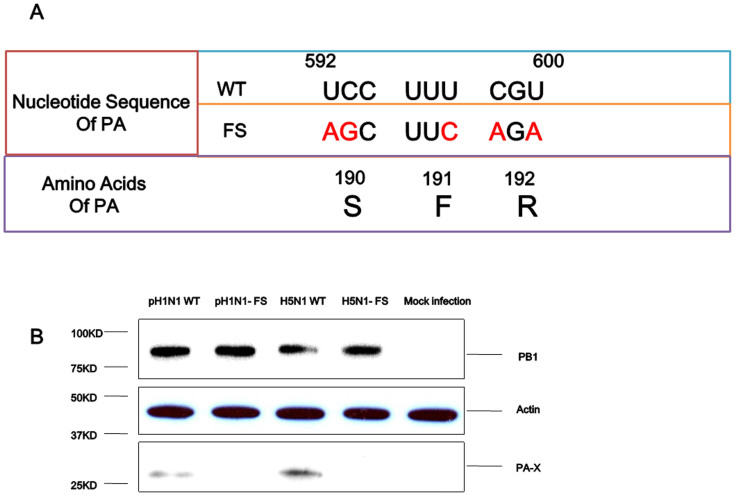
Generation of deficient PA-X in pH1N1 and HPAI H5N1 viruses. (A) Mutations (U592A, C593G, U597C, C598A and U600A in red) introduced into the PA gene to abolish PA-X expression did not alter the PA ORF. (B) Western blot analysis for the detection of PA-X protein in MDCK cells infected with pH1N1and H5N1 mutant viruses (pH1N1-FS and H5N1-FS) showed absence of PA-X unlike corresponding pH1N1 WT and H5N1 WT viruses which expressed both PA and PA-X. Mock infection served as negative control. The samples of infection cells derived from the same experiment and the blots were processed in parallel. Given the expression level of PA-X protein much lower than PB1, PA and β actin, a long exposure was used for PA-X alone. Full-length blots are presented in Supplementary Figure 2.

PA-X expression arises from ribosomal frameshift to the +1 frame at a highly conserved UCC UUU CGU sequence which is also part of the PA open reading frame (ORF)^6^. The abolition of the ribosomal frameshift site in pH1N1-FS and H5N1-FS viruses did not affect the PA ORF (Fig. 1A). Western blot analysis was performed to demonstrate the corresponding loss of PA-X expression in pH1N1-FS and H5N1-FS virus infected Madin Darby canine kidney (MDCK) cells (Fig. 1B). PA-X could be detected in pH1N1 wild type (WT) and H5N1 WT infected cells, while no PA-X was detected in cells infected with pH1N1-FS and H5N1-FS.

### PA-X deficient pH1N1 and H5N1 viruses increased virus replication and apoptosis in A549 cells

**Figure 2:**
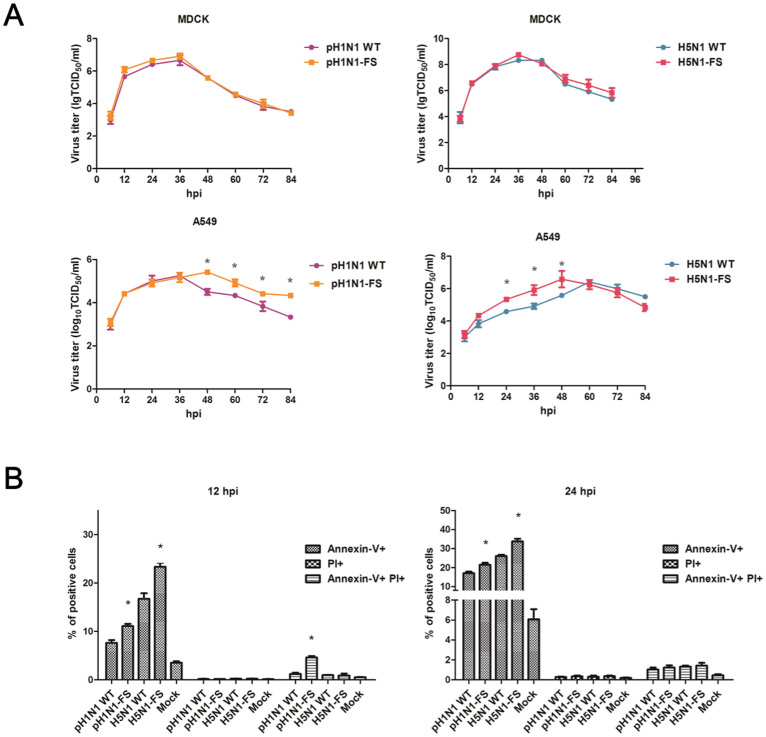
PA-X deficient viruses enhanced virus growth and apoptosis in A549 cells. (A) Virus growth curves of PA-X mutant and WT viruses in MDCK cells and A549 cells over 84 h. (B) Relative induction of cell death as determined by the detection of annexin^+^, PI^+^ and annexin^+^ PI^+^ A549 cells infected with the panel of indicated viruses at a 1.0 MOI for 12 and 24 h. * indicates significant difference between viruses with PA-X mutations and corresponding wild type viruses. Each value represents the mean of three independent experiments performed in triplicates; error bars indicate standard deviations. Differences were considered statistically significant at *P* < 0.05.

MDCK and A549 cells were infected with pH1N1 WT, pH1N1-FS, H5N1 WT and H5N1-FS viruses at 0.01 multiplicity of infection (MOI). Supernatants were collected and titrated at 6, 12, 24, 36, 48, 60, 72 and 84 hours post-infection (hpi). There was no significant difference in virus output from MDCK cells between PA-X deficient and wild type pH1N1 and H5N1 viruses (*P* < 0.05) (Fig. 2A). However, with A549 cells, viral yields of pH1N1-FS virus were higher than those of pH1N1 WT from 48 hpi (*P* < 0.05) (Fig. 2A). H5N1-FS virus also showed an increased virus output at 24, 36 and 48 hpi, but the output was reduced at 84 hpi in relation to H5N1 WT virus. Although virus titers of H5N1-FS and H5N1 WT at their peaks were similar, H5N1-FS reached the replication peak earlier. The data from A549 cells showed that PA-X deficient pH1N1 and H5N1 viruses replicated more efficiently than the corresponding wild type viruses.

Influenza viruses induce apoptosis/necrosis in infected cells as part of the resulting cellular and organ damage^15^,^16^. It has been previously shown that several viral proteins (including NS1 and PB1-F2) of influenza viruses can promote apoptosis in cells^3^,^17^,^18^. To evaluate the relative contribution of PA-X to cell death, we infected A549 cells with a panel of recombinant viruses at 1.0 MOI for 12 and 24 h. Evaluation of apoptosis and necrosis by flow cytometry for cells that were annexinV^+^ and propidium iodide (PI^+^) positive revealed that pH1N1-FS virus caused more cell death (4.62% at 12 hpi) than the pH1N1 WT virus (1.23% at 12 hpi), and evaluation of apoptosis for only annexin V^+^ cells showed that the pH1N1-FS virus produced more apoptotic cells (11.13% at 12 hpi and 21.46% at 24 hpi) than the pH1N1 WT virus (7.66% at 12 hpi and 17.03% at 24 hpi) (*P* < 0.05) (Fig. 2B). Similarly, the H5N1-FS virus produced more apoptotic cells (23.33% at 12 hpi and 33.73% at 24 hpi) than the H5N1 WT virus (16.73% at 12 hpi and 26.03% at 24 hpi) (*P* < 0.05). These results indicated that loss of PA-X expression in both pH1N1 and H5N1 viruses induced significant increases in cell death of A549 cells via apoptosis.

### PA-X deficient pH1N1 and H5N1 viruses were more virulent in mice

**Figure 3:**
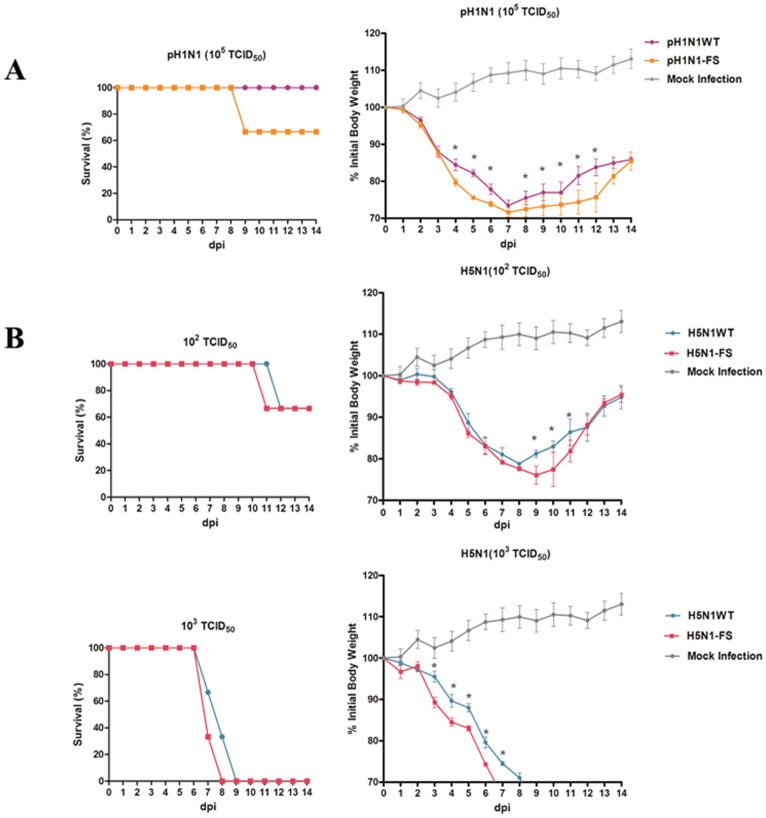
Mortality and weight loss comparisons of mice intranasally inoculated with pH1N1 (A) and H5N1 (B) viruses (PA-X deficient and wild type). Data show the survival (percentage) of mice (3 per group) infected with PA-X deficient and wild type viruses. The body weight of mice inoculated with PA-X mutant viruses was presented as percentage of the weight on the day of inoculation (day 0). Any mouse that lost more than 30% of its body weight was euthanized. The means of each group are shown, and error bars are SDs. * indicates significant difference between PA-X deficient virus and corresponding wild type virus. Differences were considered statistically significant at *P* < 0.05.

**Figure 4:**
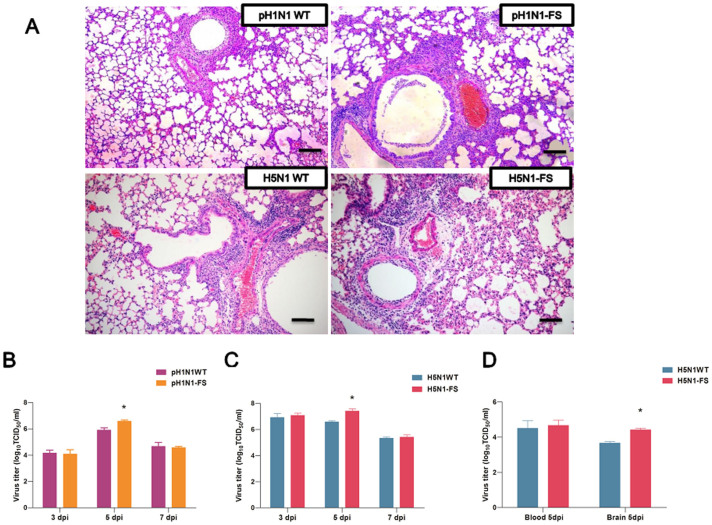
Histopathological changes (A) and virus titers in lungs (B and C) or in brains and blood (D) of mice infected with pH1N1 and H5N1 viruses (PA-X deficient and wild type). PA-X deficient pH1N1 and H5N1 viruses conferred more severe edema and inflammatory consolidation than corresponding WT viruses (A). Scale bars, 100 μm. The mean viral lung load ± SD was calculated by log_10_ TCID_50_ determination in MDCK cells. * indicates significant difference between H1N1 WT and H1N1-FS viruses, or between H5N1 WT and H5N1-FS viruses. Differences were considered statistically significant at *P* < 0.05.

To assess the pathogenicity of PA-X mutants, six-week-old BALB/c mice were intranasally infected with each virus (Fig. 3). Clinical signs, mortality and weight loss were monitored over 14 days. Virus-infected mice were humanely killed at 3, 5 and 7 days post-infection (dpi), and lungs were collected for virus titration. Survival curves at 10^5^ of 50% tissue culture infective dose (TCID_50_) clearly showed that the pH1N1-FS virus had a higher mortality rate (33.3%) than the pH1N1 WT virus with no resulting death (Fig. 3A). Virus pathogenicity was further demonstrated by histopathology of lung tissues collected at 5 dpi. As shown in Fig. 4A, the pH1N1 WT virus caused mild pathology, while moderate bronchopneumonia was observed in hematoxylin-eosin (H&E) stained lung tissues of mice inoculated with the pH1N1-FS virus. The pathological lung scores of pH1N1-WT and pH1N1-FS infected mice were 1.33 ± 0.08 and 1.83 ± 0.08 respectively. The viral titer of the pH1N1-FS virus in the lung was higher than that of the corresponding pH1N1 WT virus at 5 dpi (*P* < 0.05) (Fig. 4B).

H5N1-FS virus infected mice clearly showed greater pathogenicity than mice infected with the wild-type virus based on body weight loss and viral titers in mice (Fig. 3B). Although the 50% minimum lethal dose (MLD_50_) between mutant and WT H5N1 viruses was the same (10^2.25^ TCID_50_), body weight loss in the H5N1-FS virus group was greater, and all H5N1-FS virus infected mice died 1 day earlier at 10^3^ TCID_50_ virus dose than the corresponding H5N1 WT virus group (Fig. 3B). H5N1 WT virus infected mice showed pathological scores of 2.17 ± 0.08 and exhibited interstitial pneumonia (Fig. 4A). H5N1-FS virus infected mice, however, showed more severe interstitial pneumonia and bronchopneumonia with pathological scores of 2.67 ± 0.08, which was characterized by edema, hemorrhage, sloughing of epithelial cells and extensive infiltration of inflammatory cells. The H5N1-FS virus titer in lungs was higher than in the correspondingly derived H5N1 WT virus at 5 dpi (*P* < 0.05) (Fig. 4C). It should be noted that H5N1-FS and H5N1 WT viruses were detected in brain and blood, and that the H5N1-FS virus showed a higher titer in the brain than the H5N1 WT (*P* < 0.05) (Fig. 4D). Collectively, these infection results in mice demonstrated that pH1N1-FS and H5N1-FS viruses were more pathogenic than the corresponding wild type viruses.

### Elevated inflammatory response in mice to PA-X deficient pH1N1 and H5N1 viruses

**Figure 5:**
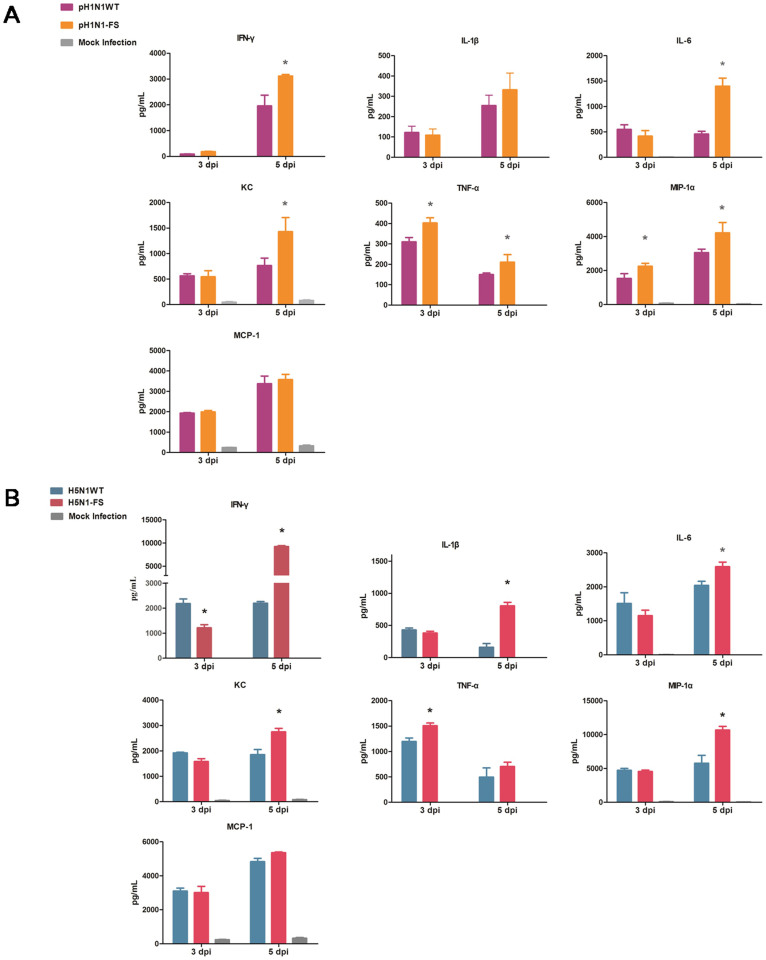
Detection of cytokine/chemokine proteins in lungs of mice infected with PA-X deficient and wild type pH1N1 (A) and H5N1 (B) viruses. Mean cytokine/chemokine levels ± SD are shown. * indicates significant difference between wild type and corresponding PA-X deficient viruses. Differences were considered statistically significant at *P* < 0.05.

It has been demonstrated that severe influenza virus infection in humans and animal models is associated with abnormally elevated pulmonary pro-inflammatory cytokine/chemokine expression^19^,^20^,^21^,^22^,^23^. To assess the effect of PA-X on host inflammatory response, we determined the protein levels of seven cytokines and chemokines in the lungs of infected mice at 3 and 5 dpi. Mice infected with the pH1N1-FS virus showed higher levels of interferon gamma (IFN-γ), interleukin-6 (IL-6), CXCL1 (KC), tumor necrosis factor-alpha (TNF-α) and macrophage inflammatory protein 1 alpha (MIP-1α) than corresponding mice infected with the WT pH1N1 virus at 3 or 5 dpi (*P* < 0.05) (Fig. 5A). Mice infected with the H5N1-FS virus also exhibited higher titers of IFN-γ, KC, TNF-α, IL-1β, IL-6 and MIP-1α than those infected with the WT H5N1 virus at 3 or 5 dpi (*P* < 0.05) (Fig. 5B). These results showed that the loss of PA-X increased the pro-inflammatory response in mice to both pH1N1 and H5N1 viruses which were consistent with the corresponding histopathological changes.

### PA-X deficient pH1N1 and H5N1 viruses increased viral polymerase activity and expression of PA protein

**Figure 6:**
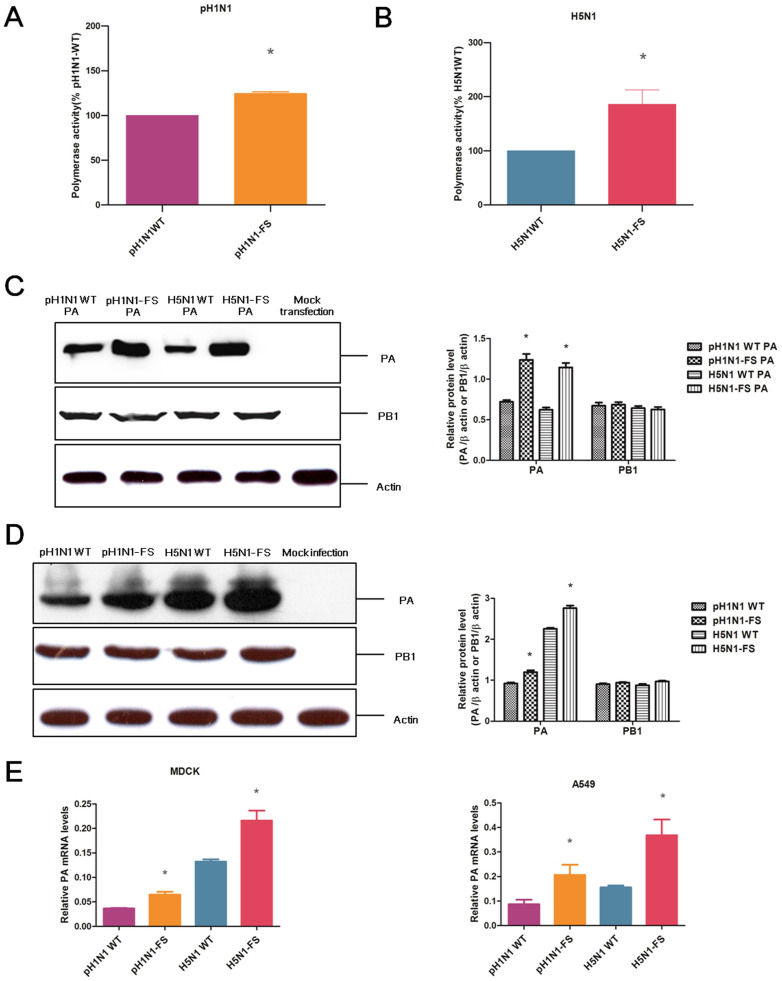
PA-X deficiency increased viral polymerase activity and expression of PA protein. Polymerase activities of PA-X deficient and wild type pH1N1 (A) and H5N1 (B) viruses are expressed as mean percentage activity ± SD, with corresponding wild-type PA set at 100% from three independent experiments. Western blot analysis to detect PA, PB1 and β-actin in protein lysates from 293T cells transfected with polymerase plasmids (C) and from MDCK cells infected with PA-X deficient and wild type viruses at 1.0 MOI for 12 h (D). The protein bands were quantified by densitometry. Relative protein levels of PA or PB1 as compared with β-actin are shown as histograms. Mock transfection and mock infection served as negative control. The samples of infection or transfection cells derived from the same experiment and the blots were processed in parallel. Full-length blots are presented in Supplementary Figure 2. MDCK cells and A549 cells (E) were infected with the indicated viruses at 1.0 MOI for 12 h. PA mRNA expression was normalized to NP mRNA expression of the same virus. * indicates significant difference between virus with PA-X deficient and corresponding wild type virus. Differences were considered statistically significant at *P* < 0.05. The values shown are means ± SD.

Ribonucleoprotein (RNP) polymerase activity has been shown to correlate with viral replication and pathogenicity^24^,^25^. We used an influenza virus mini-genome assay in 293T cells to determine the effect of PA-X on corresponding viral polymerase activity^26^. The use of PA-X deficient pH1N1 and H5N1 viruses resulted in a 24% and 85% increase, respectively, of RNP activity relative to corresponding wild type viruses (*P* < 0.05) (Fig. 6A and B).

PA-X and PA are both encoded by segment 3 of the influenza viruses; therefore, the observed increase in polymerase activity of the PA-X deficient virus might be associated with altered PA production. Western blot analysis based on protein lysates, derived from 293T cells transfected with RNP plasmids, indicated that PA protein expression was raised in the absence of PA-X for both pH1N1 and H5N1 viruses (Fig. 6C). Similarly, PA-X deficient pH1N1 and H5N1 viruses also conferred increased PA protein expression relative to the corresponding wild type virus in MDCK cells infected at 1.0 MOI for 12 h (Fig. 6D). PA mRNA expression was determined in MDCK and A549 cells infected with PA-X deficient and wild type pH1N1 and H5N1 viruses at 1.0 MOI for 12 h. PA mRNA expression (normalized to corresponding NP mRNA) from PA-X deficient pH1N1 and H5N1 viruses was higher than that of corresponding wild type viruses (P < 0.05) (Fig. 6E). In summary, abolition of PA-X protein expression increased RNP polymerase activity and accumulation of PA mRNA and protein.

### Loss of PA-X altered nuclear localization of PA protein and raised non-viral protein synthesis

**Figure 7:**
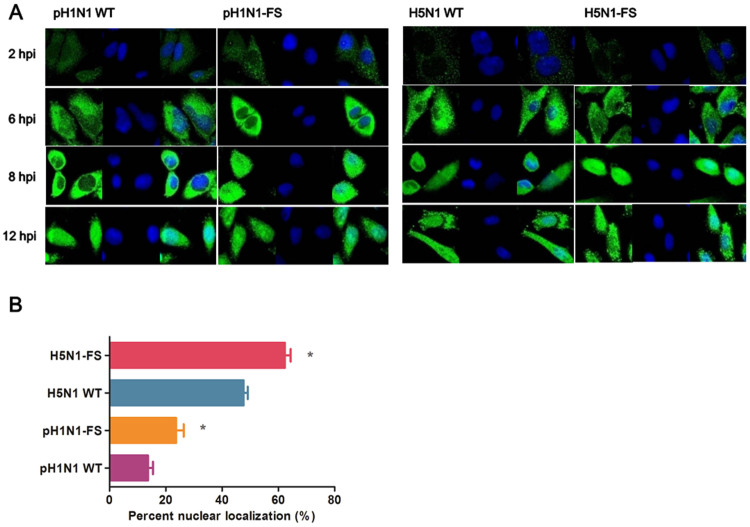
Nuclear transport of mutant and wild type PA proteins in infected cells. (A) PA protein in A549 cells infected with H1N1 WT, H1N1-FS, H5N1 WT or H5N1-FS virus at 2.0 MOI was localized by immunofluorescence at 2, 6, 8 and 12 hpi. Nuclei were stained with DAPI. (B) At 8 hpi, nuclear PA quantification was based on proportion of infected cells (n = 100) with clear nuclear presence of PA. Values shown are means of the results of three independent experiments ± SDs. *, P < 0.05 compared with corresponding wild type virus infected cells. Differences were considered statistically significant at *P* < 0.05.

Our data showed that deficient PA-X up-regulated the expression of the PA protein. Accumulation of the PA protein in the nucleus correlates with the pathogenicity of influenza viruses in mice^27^,^28^,^29^. We assessed the role of PA-X on PA protein accumulation in the nucleus of infected cells. A549 cells were infected with mutant PA and wild type pH1N1 and H5N1 viruses at 2.0 MOI. We found that in the absence of PA-X, the extension of initial cytoplasmic accumulation of PA into the nucleus was accelerated such that at 8 hpi more cells infected with PA-X deficient pH1N1 or H5N1 virus showed nuclear presence of PA than cells infected with corresponding wild type virus (Fig. 7).

**Figure 8:**
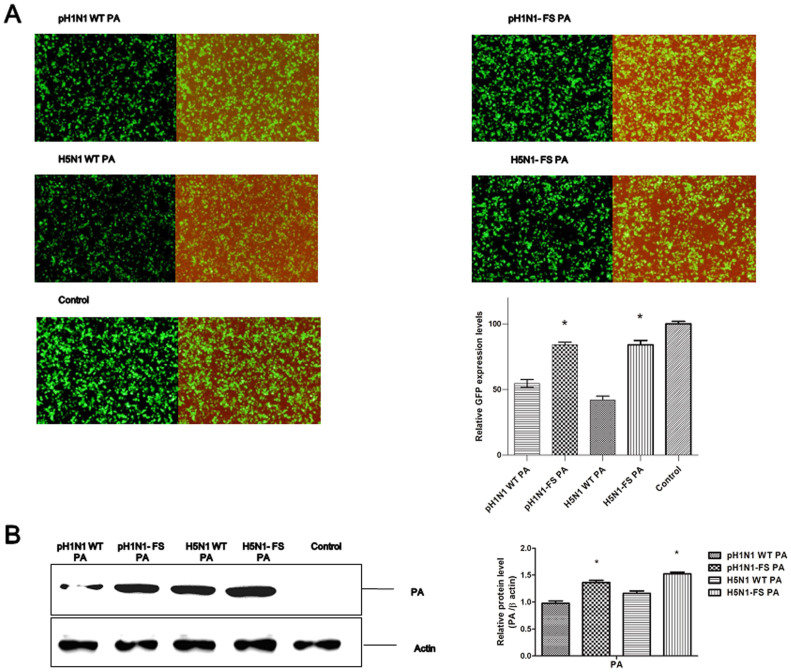
PAs were less able to suppress non-viral protein synthesis in the absence of PA-X. (A) 293T cells were co-transfected with eGFP expression plasmid and wild type or mutant PA plasmids of pH1N1 and H5N1 viruses. (A) Fluorescence images indicative of eGFP expression at 24 h post-transfection were captured under identical exposure conditions. Control was 293T cells co-transfected with eGFP expression plasmid and empty pcDNA3.1 vector. Dark and half bright field images were shown. The GFP expression levels were quantified by counting GFP positive cells by fluorescence microscopy. Relative GFP expression levels of each group as compared with control are shown as histograms. (B) Expressed PA protein was determined by Western blot analysis using anti-PA antibody. Anti-β-actin antibody was used as a loading control. The protein bands were quantified by densitometry. Relative protein levels of PA as compared with β-actin are shown as histograms. Full-length blots are presented in Supplementary Figure 2. Note PA-X deficient PA plasmids conferred more PA protein expression. Differences were considered statistically significant at *P* < 0.05. The results shown are representative data from three independent experiments. Error bars indicate standard deviations.

PA gene plays a major role in the suppression of host protein synthesis, which was partly due to PA-X function^6^,^7^. We compared the PA activities of PA-X deficient and wild type pH1N1 and H5N1 viruses in suppressing non-viral protein synthesis in 293T cells after 24 h of co-transfection with pEGFP and corresponding PA plasmids. We observed that eGFP expression was significantly higher in the presence of pH1N1-FS PA or H5N1-FS PA, compared with pH1N1 WT PA or H5N1 WT PA respectively (Fig. 8). Collectively, the absence of PA-X enhanced PA expression, accelerated nuclear accumulation of PA protein, and made PA less effective in suppressing non-viral protein expression.

## Discussion

In the present study, we evaluated the effects of PA-X in pH1N1 and H5N1 viruses on viral polymerase activity, viral replication and pathogenicity in mammalian cells and mice. We showed that loss of PA-X enhanced viral polymerase activity in 293T cells, replication in A549 cells and pathogenicity in mice for both of pH1N1 and H5N1 viruses. Jagger et al. previously showed that the PA-X deficient 1918 H1N1 virus was more pathogenic and more highly up-regulated in immune and/or inflammatory responses than the 1918 WT virus^6^. Cytokines and chemokines including IL-1β, IFN-γ, TNF-α, MIP-1α, IL-6, MIP-2, monocyte chemotactic protein 1 (MCP-1), KC and IL-1α are associated with the recruitment of macrophages and neutrophils to infected sites, which lead to acute lung inflammation^22^. We found that this spectrum of cytokines and chemokines was highly up-regulated in lungs of mice infected with PA-X deficient pH1N1 and H5N1 viruses.

In MDCK cells, there was no significant difference in viral replication between PA-X deficient and wild type pH1N1 and H5N1 viruses, which was consistent with previous findings using the PA-X deficient 1918 H1N1 virus^6^. However, in A549 cells, PA-X deficient pH1N1 and H5N1 viruses conferred increased viral replication relative to corresponding wild type viruses, with the exception of the H5N1-FS virus at 86 hpi which could be attributed to exceptionally high cell death caused by the PA-X mutant H5N1 virus. Therefore, the increase in pathogenicity of PA-X deficient pH1N1 and H5N1 viruses in mice could be the combined consequence of increased viral replication and apoptosis of infected cells coupled with enhanced inflammatory damage.

We found that loss of PA-X enhanced polymerase activity of pH1N1 and H5N1 viruses, and was accompanied by increased expression of PA mRNA and protein. We also found that PA-X suppressed expression of PA, but did not affect PB1 expression. Given that PA-X is translated from PA mRNA, we surmise that the abolition of the PA-X frameshift site promoted PA mRNA stability and translation leading to greater accumulation of both mRNA and protein, which could eventually contribute to the higher RNA polymerase activity and replication rate.

PA-X not only regulated PA expression but could also affect nuclear accumulation of the PA protein and support the suppressive role of the PA protein on host protein synthesis. Previous studies have reported that the level of polymerase accumulation in the nucleus of infected cells correlates with the virulence of the influenza virus. Huarte et al. reported that delayed PA nuclear accumulation (T157A mutation) results in reduced pathogenicity in mice^27^. Enhanced PB2 nuclear transport of H7N7 virus in A549 cells is associated with increased pathogenicity in mice^30^. Jiao Hu et al. also found that increased PA nuclear accumulation (PA K237E) is linked to virulence in ducks^29^. In contrast, Song et al. found that the delayed PA nuclear accumulation is associated with the increased pathogenicity of the H5N1 virus in ducks^28^. We found that PA-X deficient pH1N1 and H5N1 viruses showed increased nuclear accumulation of the PA protein in A549 cells at 8 hpi (Fig. 7). Enhanced PA expression and the increased nuclear accumulation of PA were consistent with elevated viral polymerase activity. In addition, in the absence of PA-X, PA appeared less able to suppress host protein expression suggesting that PA-X plays a promotional role in the suppression of host protein synthesis, which has been proposed in previous studies^6^,^7^.

In summary, our results clearly indicate that the absence of PA-X contributes to higher replication and pathogenicity in A549 cells and mice for both pH1N1 and H5N1 viruses that were associated with increased PA expression, RNP polymerase activity, and elevated inflammatory responses, thereby indicating that PA-X is a virulence modulation factor of influenza A viruses.

## Methods

### Ethics statement

All animal work was approved by the Beijing Association for Science and Technology (approval ID SYXK [Beijing] 2007-0023) and conducted in strict accordance with the Beijing Laboratory Animal Welfare and Ethics guidelines, as issued by the Beijing Administration Committee of Laboratory Animals, and in accordance with the China Agricultural University (CAU) Institutional Animal Care and Use Committee guidelines (ID: SKLAB-B-2010-005). The animal use protocol was approved by the Animal Welfare Committee of the CAU.

### Viruses and cells

The use of 2009 pandemic H1N1 [A/Beijing/16/2009 (BJ/09, pH1N1)] and HPAI H5N1 [A/Anhui/1/2005 (AH/05, H5N1)] viruses was previously described^31^,^32^. Human embryonic kidney (293T), MDCK, and human pulmonary adenocarcinoma (A549) cells were maintained in Dulbecco's modified Eagle's medium (DMEM; Life Technologies, Foster City, CA, USA) supplemented with 10% fetal bovine serum (FBS; Life Technologies), 100 units/ml of penicillin and 100 g/ml of streptomycin.

### Generation of recombinant viruses by reverse genetics

All eight gene segments were amplified by reverse transcription-PCR (RT-PCR) from BJ/09 and AH/05 viruses and cloned into the dual-promoter plasmid, pHW2000. PA-X deficient viruses, pH1N1-FS and H5N1-FS, were created by site-directed mutagenesis (QuikChange mutagenesis kit, Agilent) on the corresponding PA gene of WT pH1N1 and H5N1 viruses, which converted the frameshifting motif from UCC UUU CGU to AGC UUC AGA (U592A, C593G, U597C, C598A and U600A) to prevent the formation of PA-X^6^. PCR primer sequences are available upon request. PA ORF was unaltered in pH1N1-FS and H5N1-FS. Rescued viruses were detected using hemagglutination assays. Viral RNA was extracted and analyzed by RT-PCR, and each viral segment was sequenced to confirm the sequence identity.

All experiments with live viruses and transfectants generated by reverse genetics were performed in a biosafety level 3 containment laboratory approved by the Ministry of Agriculture of the People's Republic of China.

### Viral titration and replication kinetics

TCID_50_ was determined in MDCK cells using 10-fold serially diluted virus inoculated at 37°C and cultured for 72 h. The TCID_50_ values were calculated by the method of Reed and Muench^33^. MDCK and A549 cells were infected with viruses at a MOI of 0.01. Supernatants of the infected MDCK and A549 cells were harvested at 6, 12, 24, 36, 48, 60, 72 and 84 hpi. Virus titers were determined in MDCK cells from the TCID_50_. Three independent experiments were performed.

### Mouse infections

Fifteen mice (six week-old female BALB/c; Vital River Laboratory, Beijing, China) per group were anesthetized with Zoletil (tiletamine-zolazepam; Virbac S.A., Carros, France; 20 μg/g) and inoculated intranasally with 50 μl of 10^5^ TCID_50_ of pH1N1 or 10^3^ TCID_50_ of H5N1, diluted in phosphate-buffered saline (PBS). All mice were monitored daily for 14 days, and mice losing 30% of their original body weight were humanely euthanized. Three mice were euthanized on 3, 5 and 7 dpi for the determination of lung virus titers, histology and cytokine levels. Lungs were collected and homogenized in 1 ml of cold PBS. Virus titers were determined by TCID_50_. MLD_50_ values were determined by intranasally inoculating groups of three mice with 10-fold dilutions of each virus and were calculated by the method of Reed and Muench^33^.

### Histopathology

A portion of the lung from each euthanized mouse at 5 dpi was fixed in 10% phosphate-buffered formalin and processed for paraffin embedding. Each 5 μm section was stained with H&E and examined for histopathological changes. Lesion severity was scored by the distribution or by the extent of the lesions within the sections examined^34^ as follows: 0, no visible changes; 1+, mild focal or multifocal change; 2+, moderate multifocal change; 3+, moderate diffuse change; 4+, severe diffuse change. Two independent pathologists scored all sections from blinded experimental groups. Images were captured with a Zeiss Axioplan 2IE epifluorescence microscope.

### Viral RNP polymerase assay

RNP mini genome luciferase assays were based on the co-transfection of four pcDNA3.1 expression plasmids housing PB2, PB1, PA, and NP (125 ng each) from BJ/09 and AH/05 viruses (pH1N1 WT, pH1N1-FS, H5N1 WT, and H5N1-FS) into 293T cells together with fire-fly luciferase reporter plasmid pYH-NS1-Luci (10 ng) and internal control plasmid expressing renilla (2.5 ng). The assay was performed at 37°C. At 24 h post-transfection, cell lysates were prepared using the Dual-Luciferase Reporter Assay System (Promega) and luciferase activity was assayed using a GloMax 96 microplate luminometer (Promega).

### Quantification of cytokine/chemokine protein levels in mouse lungs

Levels of cytokines/chemokines including IL-1β, IL-6, IL-8, MCP-1, MIP-1α, TNF-α and IFN-γ in lung were determined by a cytometric bead array method (BD Cytometric BEAD Array Mouse Inflammation Kit; BD Bioscience, San Diego, CA, USA). Briefly, 50 μl mouse inflammation capture bead suspension and 50 μl detection reagent were added to an equal amount of sample and incubated in the dark for 2 h at room temperature. Subsequently, each sample was washed with 1 ml wash buffer and then centrifuged at 200 × *g* at room temperature for 5 min. Supernatants were discarded and a further 300 μl wash buffer was added. Samples were analyzed on a BD FACS Array bioanalyzer (BD Bioscience). Data were analyzed using BD CBA Software (BD Bioscience). Each chemokine or cytokine was computed as pg/ml of homogenate.

### Cell death assays

Virus infection assays were conducted in 6 well plates. Cells were seeded at a density of 1 × 10^6^ cells/well for overnight in infection media (cell growth media with 1% bovine serum albumin was used in place of FBS. Cells were then infected with virus at 1.0 MOI for 12 h. Cells from the supernatant and monolayers were then harvested, washed and stained with APC labeled annexin and PI (Becton Dickinson, San Jose, CA) for 20 min. After a final wash, cells were resuspended in 100 μl FACs wash buffer (PBS containing 3% BSA and 0.01% sodium azide) and analyzed on the FACs Calibur (BD Biosciences) and Flow Jo software (version 7.6.1). Cell death (apoptosis and necrosis) was defined as annexin-V^+^ and PI^+^, while apoptotic cells were annexin-V^+^ only. Viable cells were considered as neither annexin-V nor PI positive.

### Western blot analysis

Total cell protein lysates were extracted from transfected 293T cells or infected MDCK cells with CA630 lysis buffer (150 mM NaCl, 1% CA630 detergent, 50 mM Tris base [pH 8.0]). Cellular proteins were separated by 12% sodium dodecyl sulfate-polyacrylamide gel electrophoresis (SDS-PAGE) and transferred to a polyvinylidene difluoride (PVDF) membrane (Amersham Biosciences, Germany). Each PVDF membrane was blocked with 0.1% Tween 20 and 5% nonfat dry milk in Tris-buffered saline and subsequently incubated with a primary antibody. Primary antibodies were specific for influenza A virus PA (1:3000, GeneTex, USA), influenza A virus PB1 (diluted 1:3000, Thermo Fisher Scientific, USA), influenza A virus PA-X (diluted 1:2000, was kindly provided by Dr. Xiufan Liu, YangZhou University, China). The secondary antibody used was either horseradish peroxidase (HRP)-conjugated anti-mouse antibody or HRP-conjugated anti-rabbit antibody (diluted 1:10,000 Jackson ImmunoResearch USA), as appropriate. HRP presence was detected using a Western Lightning chemiluminescence kit (Amersham Pharmacia, Freiburg, Germany), following the manufacturer's protocol.

### RT-qPCR

Total RNA was extracted from 12 h infected MDCK and A549 cells using TRIzol reagent according to the manufacturer's instructions (Invitrogen). Oligo-(dT) primed cDNA was generated using Superscript III First-Strand Synthesis SuperMix (Invitrogen). RNA was subsequently removed by RNase A treatment for 3 h. Ten-fold serial dilutions of sample cDNA were quantified using an iScript One-Step RT-PCR with SYBR green kit (Bio-Rad). Primers used were against either PA (pH1N1 PA: Forward, 5′-AGCAGGTGCTAGCAGAGCTACA -3′; Reverse, 5′-TCACCGAGTGCCCACTTCA-3′; H5N1 PA: Forward, 5′-TGAGGCCGAGTCTTCTGTCA-3′; Reverse, 5′-CTCCACCCCTTTAGGTGATTCC-3′) or NP (pH1N1 NP: Forward, 5′-ACAATGGCGAAGATGCAACA-3′; Reverse, 5′-GTGCGAACAAGCGCTCTTG-3′; H5N1 NP: Forward, 5′-CAGCGTTCAGCCCACTTTCT-3′; Reverse, 5′-GTCAGACGTTCTGCCCTCAGTA-3′). The qPCR mixture for each sample consisted of 7.5 μl 2× SYBR green RT-PCR mixture, 5 μl of nuclease-free water, 0.6 μl of each primer, 0.3 μl iScript RNase H reverse transcriptase, hot-start iTaq DNA polymerase mixture, and 1 μl of diluted cDNA template. The qPCR samples were detected with a MyiQiCycler real-time PCR detection system (Bio-Rad), and each experiment contained three technical replicates for each sample, with two experimental replicates performed.

### Immunofluorescence

A549 cells were grown on glass-bottom dishes and infected at 2.0 MOI with the indicated virus. At specified time points post-infection, the cells were fixed with PBS containing 4% paraformaldehyde for 20 min and permeabilized with PBS containing 0.5% Triton X-100 for 30 min. After blocking with 5% BSA in PBS, the cells were incubated with rabbit antisera against PA (diluted 1:400, GeneTex, USA) at room temperature for 2 h. The cells were then washed three times with PBS and incubated for 1 h with fluorescein isothiocyanate (FITC)-coupled goat anti-rabbit secondary antibodies (diluted 1:100, Jackson ImmunoResearch, USA). The cells were subsequently washed three times with PBS and incubated with 4′, 6-diamidino-2-phenylindole (DAPI) for 10 min. Cells were imaged with a laser scanning confocal microscope (Leica). Localization of PA protein in the nucleus was determined by counting infected cells with nuclear presence of PA (n = 100). The results shown here represent three independent experiments.

### Statistical analysis

All statistical analyses were performed using GraphPad Prism Software Version 5.00 (GraphPad Software Inc., San Diego, CA, USA). The two treatment methods were compared by two-tailed Student's t-test, and multiple comparisons were carried out by two-way analysis of variance (ANOVA) considering time and virus as factors. Differences were considered statistically significant at *P* < 0.05. All data are reported as the mean ± standard deviation (SD).

## Author Contributions

J.L. designed the experiments and supervised the project. H.G., J.H., L.Q. and J.W. conducted the majority of the experimental and technical work and assisted with figure preparation. X.X., Y.W., Q.H. and Y.L. performed the comparative analysis of sequences. H.S., J.P., L.S. and W.K. assisted with data analysis, data interpretation and manuscript preparation. H.G. and Y.S. analyzed the data and wrote the manuscript. K.C., J.L., X.L., Y.S. and H.G. reviewed the manuscript.

## Supplementary Material

Supplementary Information Supplementary information
