# Supplementary material for: The contribution of PA-X to the virulence of pandemic 2009 H1N1 and highly pathogenic H5N1 avian influenza viruses
Source: Sci Rep. 2015 Feb 5;5:8262. doi: 10.1038/srep08262 (PMC4317690; doi:10.1038/srep08262)
Supplement: Supplementary Information [file srep08262-s1.pdf]

Supplementary information

The contribution of PA-X to the virulence of pandemic 2009 H1N1 and highly pathogenic H5N1 avian influenza viruses

Huijie Gao [1#], Yipeng Sun [1#], Jiao Hu [2], Lu Qi [1], Jinliang Wang [1], Xin Xiong [1], Yu Wang [1], Qiming He [1], Yang Lin [1], Weili Kong [1], Lai-Giea Seng [3], Honglei Sun [1], Juan Pu [1], Kin-Chow Chang [3], Xiufan Liu [2], and Jinhua Liu [1*]

[1] Key Laboratory of Animal Epidemiology and Zoonosis, Ministry of Agriculture, College of Veterinary Medicine, China Agricultural University, Beijing, China

[2] Animal Infectious Disease Laboratory, School of Veterinary Medicine, Yangzhou University, Yangzhou, Jiangsu Province, China

[3] School of Veterinary Medicine and Science, University of Nottingham, Sutton Bonington Campus, United Kingdom

Running title: Contribution of PA-X to virulence of pH1N1 and H5N1 viruses

[*] Corresponding author. Key Laboratory of Animal Epidemiology and Zoonosis, Ministry of Agriculture, College of Veterinary Medicine, China Agricultural University, No. 2 Yuanmingyuan West Road, Beijing 100193, China.

Tel: +86-10-62733837; Fax: +86-10-62733837; E-mail: ljh@cau.edu.cn

[#] Huijie Gao and Yipeng Sun contributed equally to this work.

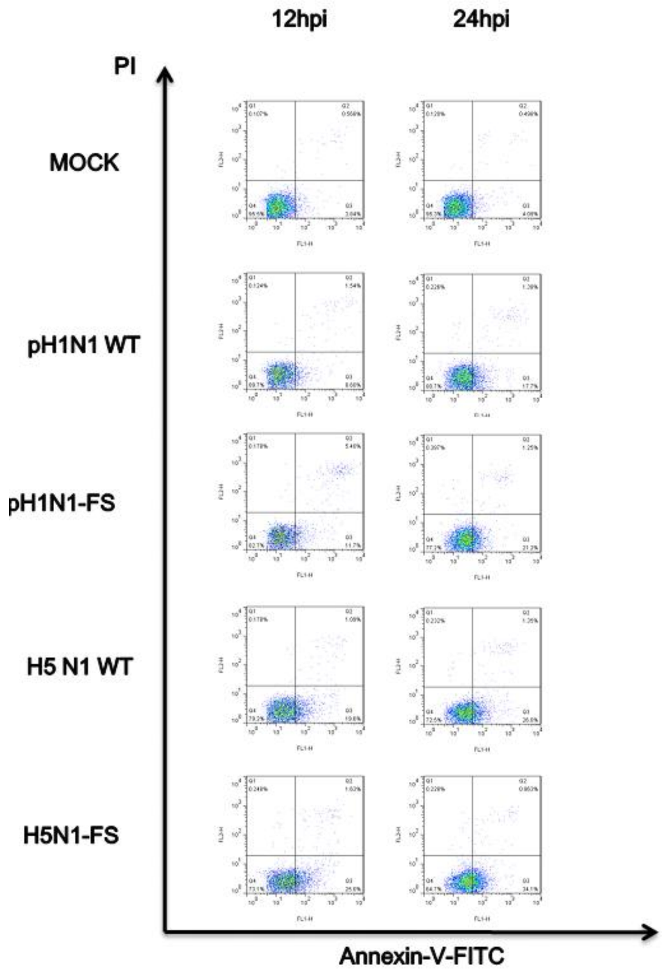

Supplementary Fig. S1. PA-X deficient viruses enhanced apoptosis in A549 cells. Relative induction of cell death as determined by the detection of annexin$^+$ and PI$^+$ A549 cells infected with the panel of indicated viruses at a 1.0 MOI for 12 hours. Representative dual-labeled quadrants of bivariant fluorescence dot plots show the relative induction of apoptosis (annexin$^+$) and necrosis (PI$^+$) in infected cells. Apoptotic cells that were positive for annexin V but not PI were identified in the right lower quadrant, and those positive for PI but not annexin V were identified in the left upper quadrant. Percentages shown are proportions of apoptotic cells. Mock, uninfected control cells.

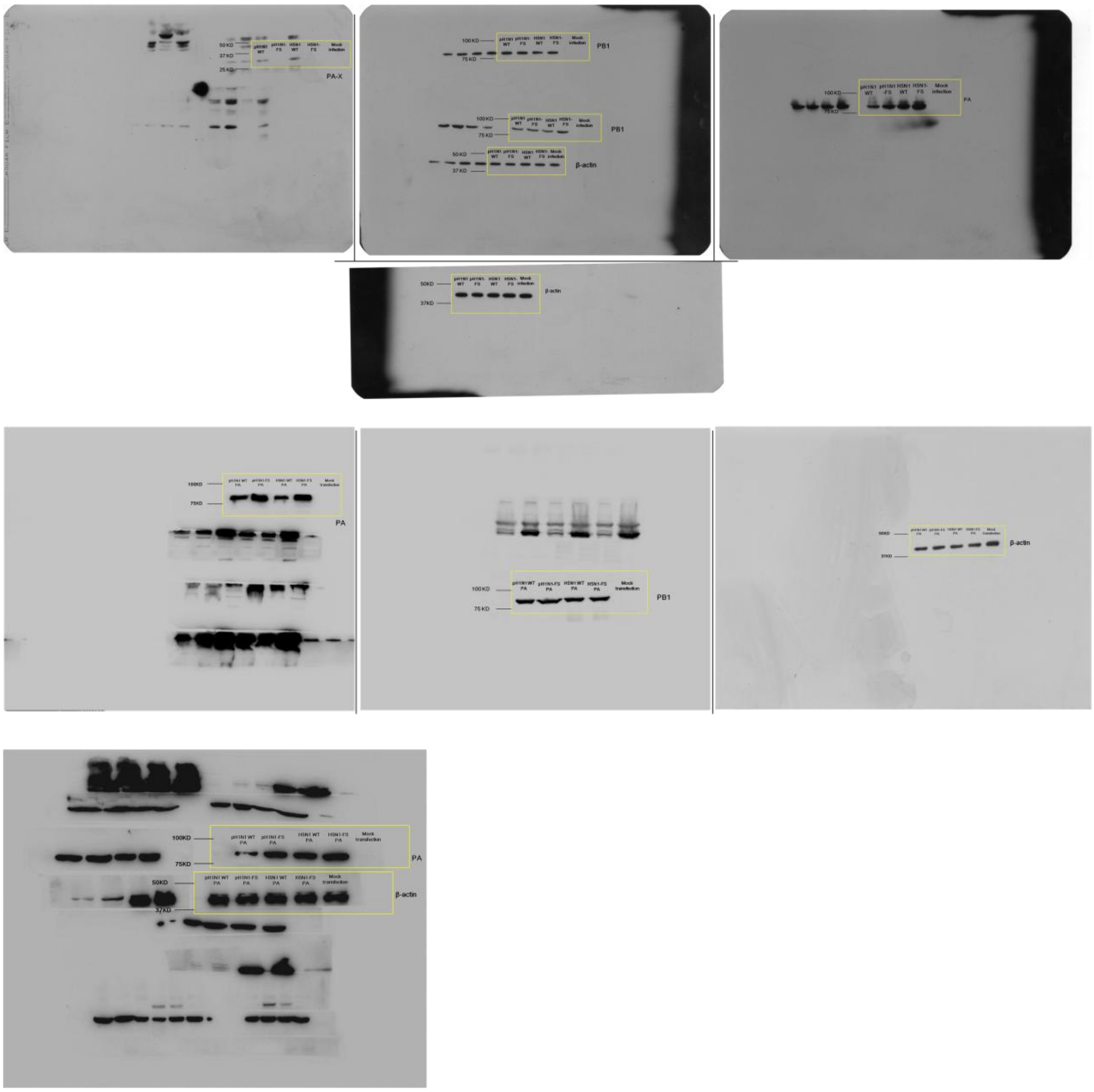

Supplementary Fig. S2. Full-length blots of figure 1B, figure 6C and D and figure 8B. Western blotting data used anti-PA-X, anti-PA, anti-PB1 and anti-β actin antibodies. Molecular weight was indicated based on the dual color marker (Bio-Rad). Because the expression level of PA-X protein is much lower than PB1, PA and β actin, a long exposure was used for PA-X alone. All the samples of infection cells or transfection cells derived from the same experiment and the blots were processed in parallel.
